# Temporal Behavioral Parameters of On-Going Gaze Encounters in a Virtual Environment

**DOI:** 10.3389/fpsyg.2021.673982

**Published:** 2021-08-06

**Authors:** Arne Hartz, Björn Guth, Mathis Jording, Kai Vogeley, Martin Schulte-Rüther

**Affiliations:** ^1^Molecular Neuroscience and Neuroimaging (INM-11), Institute of Neuroscience and Medicine, Jülich Research Center, Jülich, Germany; ^2^Translational Brain Research, Department of Child and Adolescent Psychiatry, Psychosomatics, and Psychotherapy, University Hospital RWTH Aachen, Aachen, Germany; ^3^Department of Psychiatry and Psychotherapy, University Hospital Cologne, Cologne, Germany; ^4^Cognitive Neuroscience (INM-3), Institute of Neuroscience and Medicine, Jülich Research Center, Jülich, Germany; ^5^Department of Child and Adolescent Psychiatry and Psychotherapy, University Medical Center Göttingen, Göttingen, Germany

**Keywords:** eye tracking, gaze contingency, social gaze, joint attention, ecological validity, human-agent interaction

## Abstract

To navigate the social world, humans heavily rely on gaze for non-verbal communication as it conveys information in a highly dynamic and complex, yet concise manner: For instance, humans utilize gaze effortlessly to direct and infer the attention of a possible interaction partner. Many traditional paradigms in social gaze research though rely on static ways of assessing gaze interaction, e.g., by using images or prerecorded videos as stimulus material. Emerging gaze contingent paradigms, in which algorithmically controlled virtual characters can respond flexibly to the gaze behavior of humans, provide high ecological validity. Ideally, these are based on models of human behavior which allow for precise, parameterized characterization of behavior, and should include variable interactive settings and different communicative states of the interacting agents. The present study provides a complete definition and empirical description of a behavioral parameter space of human gaze behavior in extended gaze encounters. To this end, we (i) modeled a shared 2D virtual environment on a computer screen in which a human could interact via gaze with an agent and simultaneously presented objects to create instances of joint attention and (ii) determined quantitatively the free model parameters (temporal and probabilistic) of behavior within this environment to provide a first complete, detailed description of the behavioral parameter space governing joint attention. This knowledge is essential to enable the modeling of interacting agents with a high degree of ecological validity, be it for cognitive studies or applications in human-robot interaction.

## 1. Introduction

Humans are an intensely social species and form complex social relationships. To navigate the social world, humans use not only speech but also nuanced reciprocal, nonverbal communication to initiate and respond to social encounters (Fiske and Taylor, 2013). While many of the social signals used for mutual communication are shared with other species (such as facial expression, body posture Segerstrale and Molnár, 2018, or gaze behavior Téglás et al., 2012; Catala et al., 2017), humans are experts in higher order social cognition such as inferring the intentions of a possible interaction partner (Moutoussis et al., 2014) and acting accordingly. A particularly important social signal in this respect is gaze behavior: Social gaze conveys important aspects of the inner mental state of an interaction partner (Gibson and Pick, 1963), such as his/her current focus of attention. Furthermore, social gaze constitutes a powerful communicative tool for the initiation of social contact and to signal the responsiveness for bids of social interaction. The unique morphology of the human eye (Kobayashi and Kohshima, 1997, 2001) can be considered an evolutionary imprint of the pivotal role of gaze for human communication (Emery, 2000) and its importance for shaping the phylogenetic and ontogenetic development of human social cognition (Grossmann, 2017). Thus, human gaze behavior may be an essential key to understanding human social cognition and the human mind (Shepherd, 2010).

Following a long-lasting research tradition (for early, seminal examples, see Gibson and Pick, 1963; Kendon, 1967; Yarbus, 1967; Argyle and Cook, 1976; Kleinke, 1986) experimental paradigms in social gaze research have typically relied on static images or prerecorded videos as stimuli. However, in the light of the complex and dynamical character of nonverbal communication (Burgoon and Buller, 1989; Krämer, 2008; Vogeley and Bente, 2010), true interactionist approaches call for higher ecological validity (Risko et al., 2012, 2016; Pfeiffer et al., 2013a; Schilbach et al., 2013). Of particular interest in contemporary social gaze research is the dynamic, reciprocal gaze behavior of two interacting agents during social interaction, as evident, for example during instances of joint attention (JA) (Moore et al., 2014). JA is characterized by a shared attentional focus of two people on an object (Emery, 2000, commonly also referred to as “triadic interaction”). More recently, gaze-contingent paradigms have been developed to investigate the dynamic aspect of JA (e.g., Wilms et al., 2010; Pfeiffer et al., 2014; Oberwelland et al., 2016, 2017, for a review see Pfeiffer et al., 2013b). However, even these gaze-contingent approaches have not yet considered or modeled extended periods of unfolding interactions. They are often restricted to explicitly instructed, “atomic units” of interaction, e.g., single gaze shifts without acknowledgment of their embeddedness in and governance by higher-order mental states of the interacting agent(s). In this respect, empirical investigations face an inevitable dilemma. On the one hand, two interacting humans could be studied instead, resulting in enhanced ecological validity but lacking the type of controlled experimental manipulation which is desirable for cognitive experiments. Otherwise, if experiments are confined to predefined and scripted units of behavior, they neglect the dynamic and highly reciprocal character of social interaction. An essential step to escape this dilemma is therefore to develop a rich parameterized model of gaze behavior in a virtual human-like agent which provides full flexibility to study dynamic interaction, but provides complete control over its behavior at the same time.

Previous work has shown that it is possible to create virtual agents with credible JA capabilities (see e.g., Wilms et al., 2010; Courgeon et al., 2014; Grynszpan et al., 2017), however, these approaches mostly focused on specific aspects of social gaze interaction or visual attention (e.g., Hoekstra et al., 2007). It is of particular importance to ground such attempts within an adequate conceptual framework and to provide a situation-specific taxonomy and exhaustive description of the behavior of interest. We have proposed the concept of “*Social Gaze States*” (Jording et al., 2018) which provides a comprehensive description of the space of possible states during encounters between two agents and an object in a shared environment. It introduces interactive and non-interactive states: An agent may attempt (a) to initiate joint attention (*IJA* state) by shifting its gaze toward an object and expecting a corresponding shift of the other agent's gaze to the same object or (b) to respond to a respective joint attention bid of the other agent (*RJA* state). With respect to non-interactive states (no gaze-contingent response to the other agent's gaze behavior), we distinguish an object-oriented (*OO*) state (i.e., the agent is focused on an object), a partner-oriented (*PO*) state (i.e., the agent is focused on the other agent), and an introspective (*INT*) state (i.e., the agent is neither focused on the other agent, nor an object).

The present paper aims to provide the implementation of an algorithmically controlled agent mimicking the full range of behavior as conceptualized in our concept of Social Gaze States within a highly controlled, yet flexible, gaze-contingent experimental environment. To this end, we determine the free temporal and probabilistic model parameters implied by this framework and demonstrate how it can be used to investigate the temporal and probabilistic dynamics of human social gaze interaction.

## 2. Methods

This section first describes the algorithmic modeling of the behavior of a virtual agent according to the theoretical concept of the Social Gaze States during gaze-based interactions between two agents and an object. Second, we demonstrate how this model can be used to empirically determine temporal and probabilistic parameters of such interactions in humans.

### 2.1. Modeling of the Agent's Gaze Behavior

#### 2.1.1. Experimental Environment

**Figure 1:**
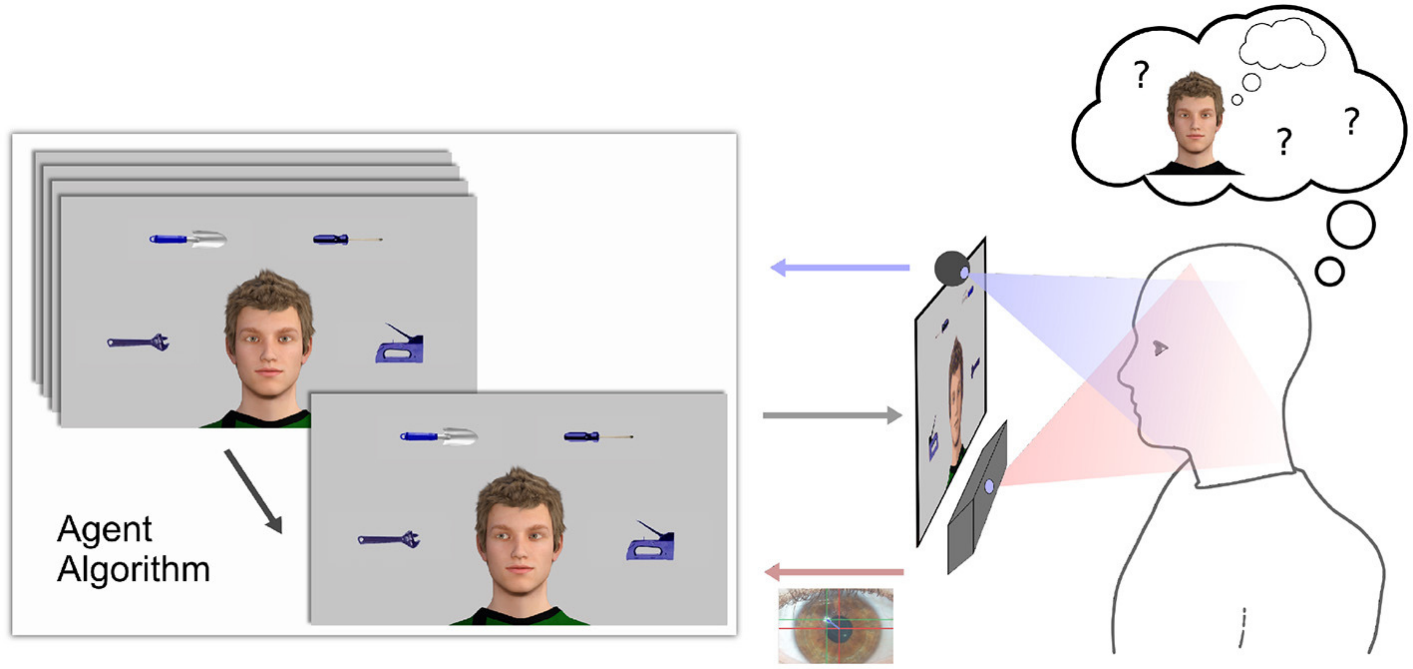
Technological architecture: Eye tracker and video camera for data acquisition, computer screen for stimulus presentation, and computer for gaze-contingent agent algorithm, system integration, and data collection.

The facial display of a virtual agent is located at the center of a computer screen. It is surrounded by objects and can interact with a human participant by means of eye movements (Figure 1).

#### 2.1.2. Social Gazes States (Macro States)

Conceptually, we distinguish macro states (i.e., higher order mental states of the agent), and micro states (i.e., the concrete building blocks of behavior manifesting as gaze shifts). The set of macro states in a given experiment spans the space of all possible states of the agent in the respective setting and are defined according to theoretical considerations as laid out in the Social Gaze Space taxonomy. A macro state is a relatively persistent state which is represented to the human by a algorithmically generated sequence of shorter “micro states.” In the specific use-case described here, the macro states are given by the concept of Social Gaze States, i.e., interactive states (*IJA*, *RJA*), and non-interactive states (*PO, OO, INT*) (Jording et al., 2018). Note, these states have a straightforward, simple definition, but can still be considered as higher-order mental states because they contain the absence of intentional social interaction.

#### 2.1.3. Individual Gaze Shifts (Micro States)

Each macro state is composed of several micro states. The temporal dynamics of a macro state are governed by (sometimes gaze-contingent) transitions between well-defined micro states and their durations. Importantly, to avoid the impression of deterministic behavior, timing and choice are defined probabilistically. The next action (i.e., a gaze shift to a specific location) is determined by transition probabilities between micro states, whereas the timing (i.e., reaction time to social-emotional signals or dwell times on specific locations) is determined by a draw from a respective reaction time distribution. This implementation renders an agent's behavior much more natural and comparable to human behavior. Thus, a micro state is defined by (1) its visual appearance (i.e., gaze direction), (2) its duration (drawn from a state specific random distribution) (3) transition probabilities (with respect to all other micro states entailed in the current macro state) (4) sensitivity to gaze signals of the human [i.e., fixation on areas of interest (AOI), which in turn may trigger a transition to a different micro state].

For a formal definition, see Supplementary Material (section 1.1).

#### 2.1.4. Interactive Macro States

Interactive macro states contain micro states that are sensitive to gaze cues. This section describes the implementation of two specific types of gaze-contingent macro states, namely responding to joint attention (*RJA*) and initiating joint attention (*IJA*). Again, macro states are characterized by probabilistic choices of the next action (i.e., gaze shift) of the agent and specific timing distributions (i.e., either for moving to the next micro state or reacting to a socio-emotional signal of the human).

The *RJA* macro state is characterized by the agent following the human's gaze. When the human fixates any object or looks straight at the agent, the agent follows with its eyes, thus either jointly looking at the same object or establishing eye-contact, with a specific probability and temporal delay. Then, the agent will keep fixating the AOI for a specific duration before it is ready to follow again. (Formal state diagram: Supplementary Figure 2; formal micro state definitions: Supplementary Table 1).

The *IJA* macro state is characterized by the agent attempting to initiate JA with the human on an object. First, the agent gazes straight waiting for eye contact or a given maximum duration before shifting its gaze toward an object with a specific probability. Next, if the gaze shift was followed by the human within a given delay or if no JA occurred, the agent gazes straight back to the human (after a given delay) trying to reestablish eye contact to commence another JA bid. (For a formal state diagram: Formal state diagram: Supplementary Figure 3; formal micro state definitions: Supplementary Table 2).

#### 2.1.5. Non-interactive Macro States

Within our framework, the object- and partner-oriented as well as the introspective macro states are implemented as non-interactive. State transitions are generated by a simple generic Markovian process (i.e., the next micro state is only dependent on the current micro state). This is implemented by modulating the probabilities for transitions between micro states: For example in the *OO* state, the probability for a transition to a micro state with focus on an object is much higher than in the *PO* state. This creates the impression of a specific attentional focus [e.g., to objects (*OO*) or the partner (*PO*)] of the agent. During the *INT* state only very few gaze shifts toward objects or the interaction partner occur (suggesting inwards directed attention, referred to as “introspection”). Furthermore, transitions between micro states are determined by temporal parameters, e.g., the time spent on fixating an object or the interaction partner. (Formal state diagram: Supplementary Figure 4; formal micro state definitions: Supplementary Table 3).

#### 2.1.6. Other Behavioral Aspects

The descriptions above represent the core implementation of the concept of the Social Gaze States. In addition, further features can be used for conceptual extensions and refinements: It is possible to implement aspects of behavior that are not dependent on the specific macro states, but consistently displayed during face-to-face interactions (“superimposed”). As a prototype, eye blink behavior is implemented by transitioning shortly (100 ms) to a micro state with closed eyes, and than transitioning back to the previous micro state. The time interval between two blinks is drawn from a random distribution with associated parameters analog to the micro state duration detailed above (see Supplementary Videos).

Our conceptualization of micro and macro states allows for a broad range of possible implementations of agent behavior in triadic settings by simply creating, modifying, and (re)combining micro states via transition rules. For example, emotional expressions can easily be added to the framework by defining additional micro states with the agent displaying facial emotions. Thus, it is possible to generate social scenarios which correspond to a range of more complex higher order mental states and socio-emotional situations.

#### 2.1.7. Additional Features and Software Implementation

We also provide availability of real-time classification of facial expression of the participant. A Python client for the FaceReader API (Noldus Information Technology, The Netherlands, tested for Versions 6.X and 7.X, available for Microsoft Windows only) is integrated and allows for emotion-contingent settings. For automated, synchronized video recordings of the participant during the experiment, support for video capturing via μCap (Doyle and Schindler, 2015) is implemented. This allows for synchronized offline analysis of video recordings of the experimental procedure, e.g., for in-depth offline facial action unit activity and emotion of the participant (Friesen and Ekman, 1978; Schulte-Rüther et al., 2017).

The framework supports arbitrary AOI definitions, allowing e.g., for fine-grained AOI definitions of different aspects of the face, or the displayed objects. The virtual environment is implemented in Python 2.7 (Python Software Foundation, https://www.python.org/) based on the open-source package(s) PyGaze (Dalmaijer et al., 2014) acting as a wrapper for PsychoPy (Peirce, 2009) for stimulus presentation and eye tracker integration. Our current implementation uses the Software Development Kit (SDK) for Tobii Eye trackers.

Further details of hard- and software requirements, dependencies, toolbox layout, and technical reliability can be found in Supplementary Material (section 1.2).

### 2.2. Empirical Investigation

Next, this framework was used to empirically determine the temporal and probabilistic dynamics of the described Social Gaze States in ongoing interactions between an agent and a human participant. To this end, timing and probabilities for gaze shifts were measured and corresponding distributions were estimated.

#### 2.2.1. A-Priori Parameters

For a given experiment, the parameters defining each of the agent's micro states must be set *a-priori*. In a first approximation, all distributions were defined as Gaussians and numerical parameter values were chosen based on a-priori knowledge (Pfeiffer et al., 2012; Oberwelland et al., 2016, 2017; Willemse et al., 2018) and refined using a face-validity strategy such that the desired behavior of the agents appeared “natural” based on intuitive judgments by the authors. These parameters defined the behavior of the agent that was used in the present empirical study (Supplementary Tables 7, 10). Throughout the experiment the agent displayed eye blinks at a mean rate of 17 blinks per minute.

#### 2.2.2. Paradigm

Thirty-seven (four excluded) adult participants without any past medical history of neurological or mental disorders were asked to interact with the algorithmically controlled agent in 60 blocks of ~ 30 s each. The experiment was divided into two parts with a short break in between to give participants the opportunity to relax and to prevent drifts in eye tracking measurements by recalibration. For details on the recruitment procedure, see Supplementary Material (section 1.3.1).

Before each block, participants were instructed via screen messages (Supplementary Table 4) to explicitly show behavior of the five Social Gaze States (Jording et al., 2018). State order was assigned randomly, evenly balanced across the course of the experiment (Supplementary Table 6).

When participants were asked to engage in the non-interactive states (*PO, OO, INT*), the agent either predominantly gazed directly at them (*PO* state) or mainly shifted its gaze downwards and avoided eye contact (*INT* state). State transitions occurred independent of the participant. When the participant was instructed to follow the agent's gaze (*RJA*) the agent was always in the *IJA* state. When the participant was asked to initiate interactions (*IJA*), the agent was put in one of two *RJA* states which differed in their probability to follow the participant's gaze shifts (either *RJA*_*high*_, i.e., agent always following ($p_{f}^{RJA1}=1.$), or *RJA*_*low*_, i.e., following with probability $p_{f}^{RJA2}=0.33$) in order to measure behavioral parameters when JA bids are not followed by the agent. For each block, four images of household items (adapted from Bayliss et al., 2006) were randomly chosen and displayed at given screen positions (Figure 1, Supplementary Figure 5).

#### 2.2.3. Estimation of Temporal Parameters

**Figure 2:**
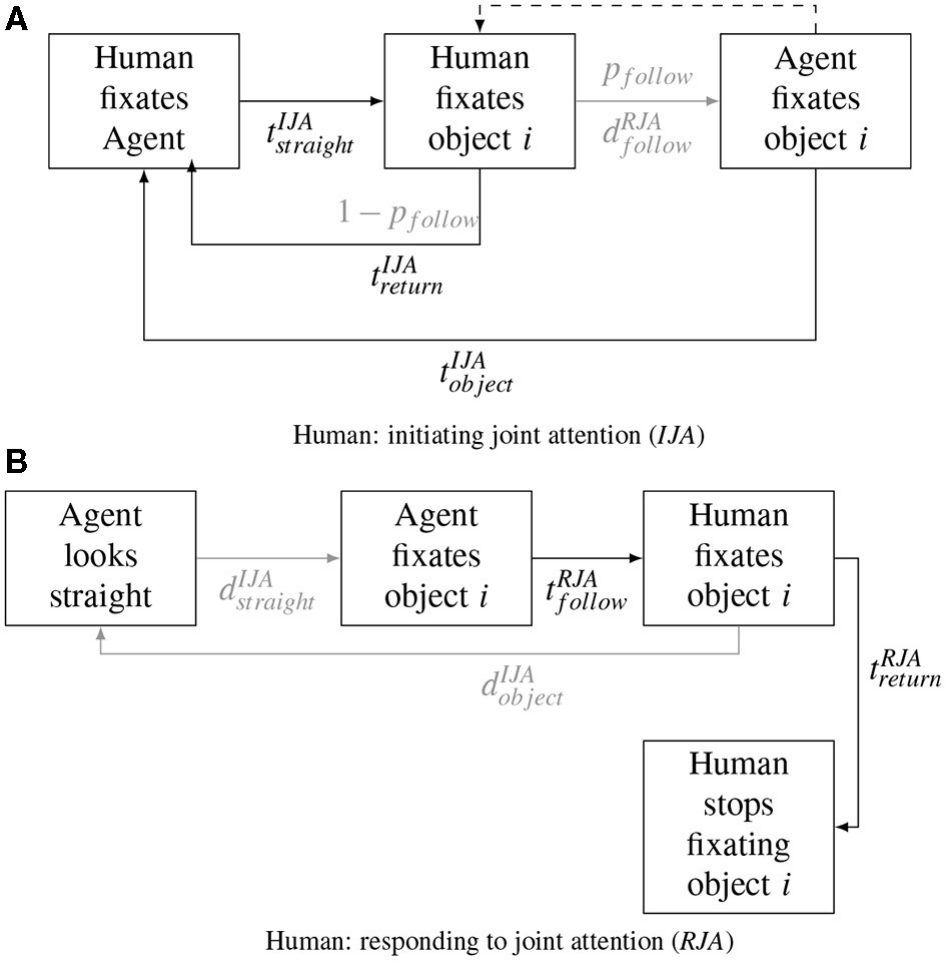
Sequences of behavioral events during the interaction between a human and the algorithmically controlled agent. **(A)** The human initiates joint attention (JA) and the agent responds. **(B)** The agent initiates JA and the human responds. *ts* denote dwell times and reaction times of the human, *ds* denote dwell times and reaction times of the agent. Note, that **(A)** allows for two possible variants of behavior for the human after gazing at an object, i.e., either fixating the agent again before trying to initiate JA on new object ($t_{r}^{IJA}$), or directly fixating the next object without gazing back to the agent (dashed line).

Following the definitions of agent behavior, reaction times (RT) and dwell times (DT) were defined as temporal parameters characterizing the interactive behavior of the participant. RT refers to the time between the onset of an agent's micro state (i.e., gaze shift on an object) and the response of the participant (i.e., fixation on the same object) (see e.g., Figure 2). DT refers to the time between the onset of the first and the end of the last of all consecutive fixations on an AOI by the human ignoring micro saccades within AOIs.

In order to assess the natural variance of RTs and DTs we fitted probabilistic distributions typically used to model human behavior (Normal (norm), Log-normal (lnorm) (Limpert et al., 2001), and exponentially modified Normal distribution (ExGauss) Ratcliff, 1979), separately to each participant's data (fitdist() from the R package fitdistrplus v1.0.9; Delignette-Muller et al., 2015). The best fitting distribution *q* was selected via the Bayesian information criterion (BIC) and, for this selected distribution, distribution parameters Θ were averaged across participants.

Further details on data exclusion and preprocessing are detailed in Supplementary Material (section 1.4).

#### 2.2.4. Micro State Transition Probabilities

Similarly, transitions probabilities from one micro state to the other were determined empirically based on the observed frequency of gaze shifts between the respective AOIs. Values were computed for each participant and subsequently averaged.

#### 2.2.5. Verification of Gaze-State Induction

In order to verify the successful induction of gaze states we assessed separately for each state the participant's allocation of attention by calculating fixation heatmaps and dwell times for different AOIs.

Fixation heatmaps were constructed by drawing circles around each fixation position convoluted with a quadratic density distribution and with a radius equivalent to the standard deviation between individual gaze data position. These values (now proportional to the overall duration of fixations) were subsequently logarithmization for optimized illustration (see Supplementary Material for details).

On-AOI ratios were defined as the ratio of the time of the participant dwelling on any of the defined AOIs in a macro state *M* over to the total state duration. It served as a measure of to what extent the participants attention was focused within the virtual environment during the experiment.

(1) $$\begin{array}{r} r_{aoi}^{M}=\frac{\sum DT^{M}}{duration\;state\;M}\leq 1 \end{array}$$

Face-object ratios were defined as the ratio of the time the participant spent dwelling on the agent compared to the objects in each macro state *M*. It served as a measure of how the attention of the participant was distributed between the objects and the agent during the experiment.

(2) $$\begin{array}{r} r_{a/o}^{M}=\frac{\sum DT_{agent}^{M}}{\sum DT_{agent}^{M}+\sum DT_{object}^{M}}\leq 1 \end{array}$$

We used one-way repeated measures ANOVAs to compare the face-object ratio across the induced gaze states.

## 3. Results

Due to general calibration problems of the eye tracker, four participants had to be excluded entirely from further analysis. Of a total of 2,188 blocks, 38 had to be excluded due to a bug in the presentation of instructions to the participants. Due to a bug in the agent's eye-blinking behavior 184 blocks had to be excluded. Both bugs are fixed in the release version. Another 419 blocks were excluded, because <66.6% of eye tracking data was available for each of theses blocks (e.g., head movement outside the tracking box of the Tobii system, and participants wearing glasses incompatible with the Tobii system). This leaves a total of 1,547 blocks included in data analysis (71%).

### 3.1. Parameters Indicating the Successful Induction of Social Gaze States

**Figure 3:**
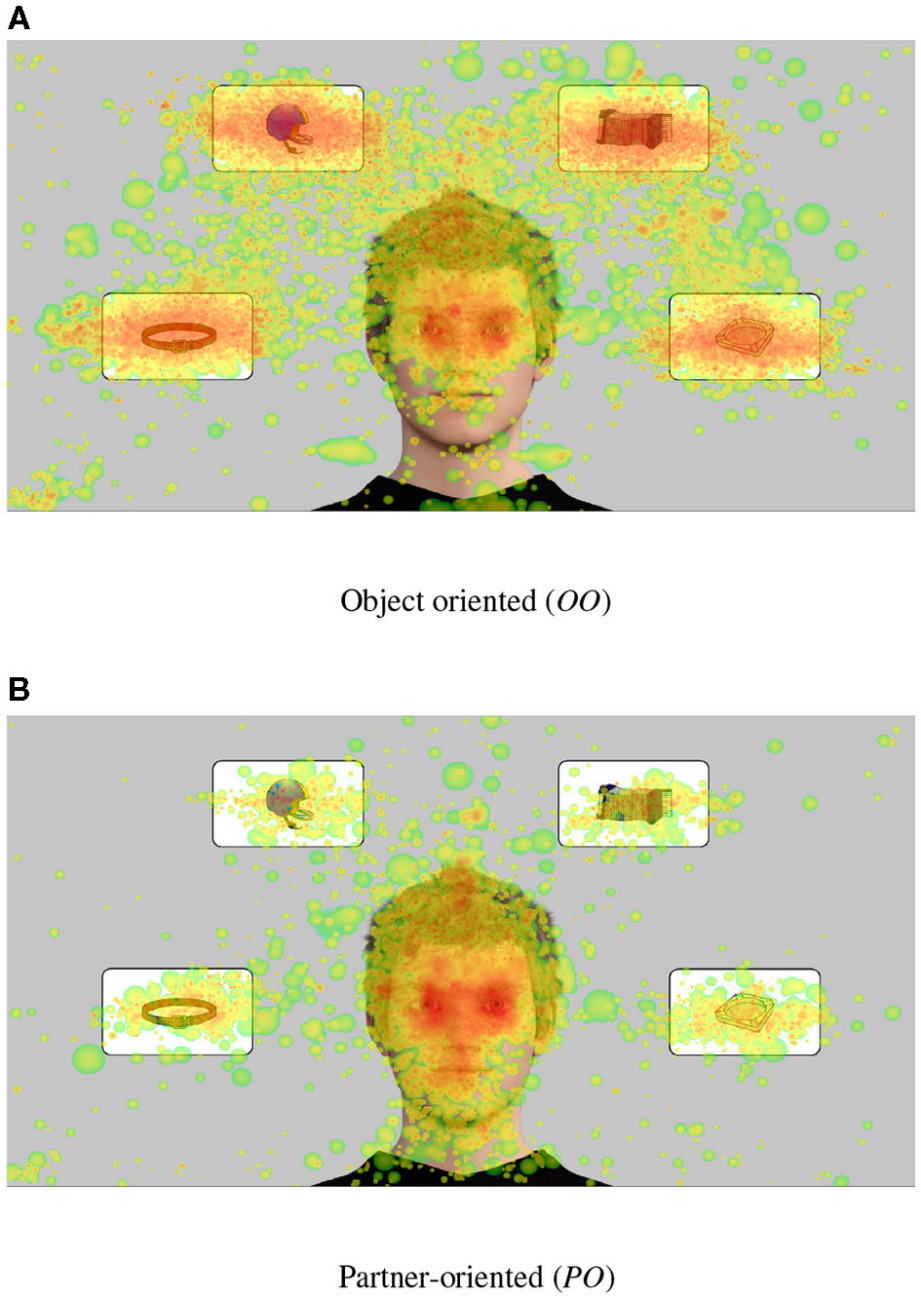
Aggregated fixation heat maps for all participants in the **(A)** object-oriented (*OO*) and **(B)** partner-oriented (*PO*) states. For quantitative values see Figure 4. The small cluster in the lower left corner corresponds to the position of the stimtracker sensor on the screen.

**Figure 4:**
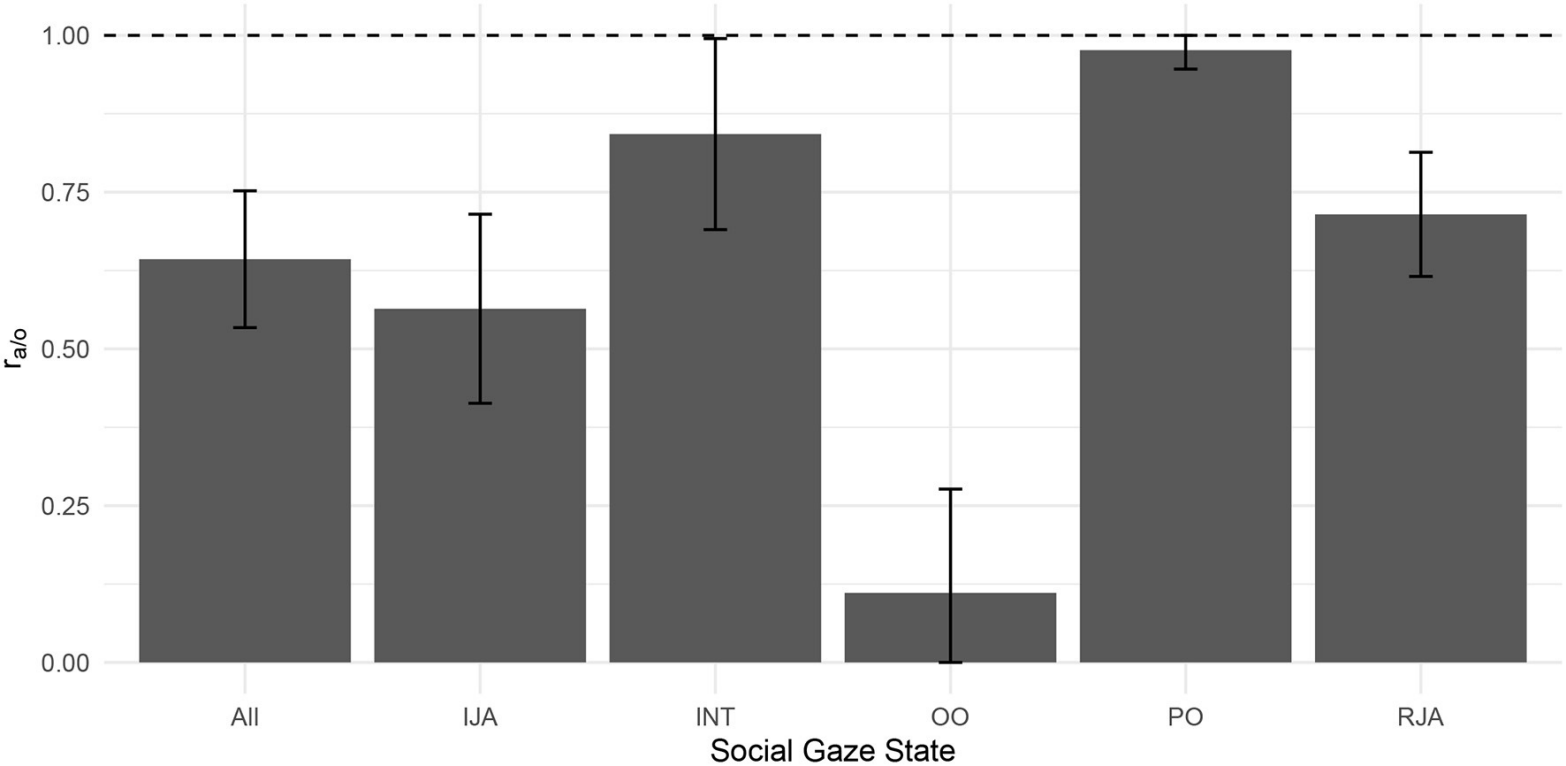
Average face-object ratio $r_{a/o}^{M}$ across participants for the Social Gaze States. Error bars indicating SD (capped at 0 and 1).

Fixation heatmaps for the *OO* and *PO* states combined for all participants demonstrate a qualitative difference in participants' gaze behavior (Figure 3). When statistically comparing all gaze states across participants, a one-way repeated measures ANOVA [*F* = 8.72, *f* = (4, 124), *p* < 0.01, η^2^ = 0.22] revealed that the fraction of time spent dwelling on the agent's face in comparison to the objects (*r*_*a*/*o*_, see also Figure 4) was different across gaze states. *Post-hoc t*-tests revealed significant differences for pair-wise comparisons of all conditions [all *T*(64)>5.24, all *p* < 0.01, Bonferroni-corrected for multiple comparisons], with the biggest difference between the partner-oriented and object-oriented states [*T*(64) = 26.4].

Participants spent most of the time during the experiment either fixating one of the four objects or the agent 〈_*r*_*aoi*_〉*human*_ = 0.84(±0.07 SD) (Supplementary Figure 7), indicating that they focused their attention mostly within the virtual environment.

The agent made attempts to initiate JA with a frequency of 18(±4*SD*) per minute, which is equivalent to 112 trials per participant on average. Participants responded with a gaze shift toward the same object on average in 85(±20*SD*)% of the cases. With about the same frequency (15±7 per minute), the participants made attempts to initiate JA when asked to lead the gaze, which is equivalent to 96 trials per participant.

Overall, these results indicate that participants followed the instructions and the Social Gaze States could reliably be induced and measured.

### 3.2. Empirical Behavioral Parameters

#### 3.2.1. Temporal Parameters

Means and standard deviations of all RTs and DTs (as well as the corresponding distribution and defining parameters) are reported in Table 1.

**Table 1:** Temporal behavioral parameters estimated from behavioral sequences as shown in Figure 2.

**Macro state**	**Timing parameter**	**Description**	**Mean**	**SD**	**Estimated distribution**	**μ**	**ρ**	**η**	**Est. mean**	**Est. SD**
IJA	$t_{straight}^{IJA}$	DT on partner before trying to initiate JA	985	584	lnorm	6.72	0.46	–	979	580
	$t_{object}^{IJA}$	RT after successful JA before gazing back at partner again	888	409	lnorm	6.50	0.58	–	917	566
	$t_{back}^{IJA}$	DT on object when trying to initiate JA, but partner is not following	1590	665	lnorm	7.26	0.44	–	1,540	702
RJA	$t_{follow}^{RJA}$	RT after which agent follows partner on AOI	463	133	exGAUS	350	34.20	124	474	130
	$t_{object}^{RJA}$	DT on object after JA was successfully initiated	995	290	lnorm	6.84	0.27	–	986	294
PO	$t_{object}^{PO}$	DT on objects	435	280	lnorm	5.92	0.53	–	482	342
	$t_{agent}^{PO}$	DT on face	1,810	1,600	norm	2,480	1,670	–	1,860	1640
OO	$t_{object}^{OO}$	DT on objects	1,430	1,140	exGAUS	566	358	824	1,390	968
	$t_{agent}^{OO}$	DT on face	660	612	lnorm	6.27	0.67	–	662	756
INT	$t_{object}^{INT}$	DT on objects	687	628	lnorm	6.04	0.59	–	710	665
	$t_{agent}^{INT}$	DT on face	1,870	1,690	lnorm	7.27	1.04	–	2,060	3,030

*Mean and standard deviation (SD) of dwell times (DT) and reaction times (RT), best fitting distribution (determined via Bayesian Information Criterion), and associated parameter sets {μ, ρ, η}. Estimated mean (est.mean) and SD (est.SD) were calculated using the fitted parameter sets. IJA, Initiating joint attention; RJA, responding to joint attention; PO, partner-oriented; OO, object-oriented; INT: introspective*.

#### 3.2.2. State Transition Probabilities

Empirical transition matrices for exemplary defined AOIs (one for each object, one for the agent, see Supplementary Figure 7) for the *OO* and *PO* states are presented in Tables 2, 3. Higher transition probabilities toward object AOIs compared to the facial AOI in the *OO* state are compatible with the higher proportion of object DT. Object AOIs that were displayed in proximity have higher transition probabilities, suggesting mostly sequential exploring of adjacent objects.

**Table 2:** Estimated transition probabilities for the *OO* state.

**AOI**	***O*_1_**	***O*_2_**	***O*_3_**	***O*_4_**	***A***
*O*_1_ (left)	–	0.65	0.08	0.09	0.15
*O*_2_ (up left)	0.38	–	0.50	0.04	0.08
*O*_3_ (right)	0.05	0.45	–	0.43	0.08
*O*_4_ (up right)	0.15	0.10	0.55	–	0.23
*A* (straight)	0.28	0.31	0.19	0.21	–

*Transition probabilities were calculated for shifting gaze from an object or the agent (O i, A; row) to a different target (O j, A; column) during the OO state. For more details see Supplementary Material (section 1.1)*.

**Table 3:** Estimated transition probabilities for the *PO* state.

**AOI**	***O*_1_**	***O*_2_**	***O*_3_**	***O*_4_**	***A***
*O*_1_ (left)	–	0.17	0.01	0.06	0.76
*O*_2_ (up left)	0.27	–	0.42	0.02	0.29
*O*_3_ (right)	0.00	0.33	–	0.21	0.46
*O*_4_ (up right)	0.03	0.01	0.12	–	0.84
*A* (straight)	0.35	0.15	0.07	0.43	–

## 4. Discussion

The present implementation provides a new experimental platform for a highly controlled fine-grained quantitative investigation of human social gaze behavior in joint attention settings. We showcase the implementation of a theoretical model and taxonomy of gaze-based interaction, i.e., socio-emotional states spanning a “Social Gaze Space” (Jording et al., 2018). However, the platform is not restricted to this particular concept of the Social Gaze States, but is flexible and can accommodate further socio-emotional behavioral modalities for future extensions or adaptations of the model. Promising applications include (1) the facilitation of naturalistic human-agent (either virtual or robotic) interaction and (2) social gaze behavior and its deviations in psychiatric conditions. In both cases, information on the exact timing in reciprocal social gaze behavior is crucial.

### 4.1. Social Gaze State Parameters

The present framework provides the algorithmic implementation of our taxonomy of Social Gaze States (Jording et al., 2018). We used a virtual agent to embody our model and tested and refined a-priori considerations during real-time human interaction. Importantly, we were able to determine probabilistic and temporal parameters for typical human gaze behavior using empirical data, which is relevant for the concept of the Social Gaze States and beyond. To our knowledge, this is the first study providing a complete description of the behavioral parameter space for ongoing gaze-based interactions during JA. The findings are in line with reports from the few previous studies investigating receptive and interactive gaze.

Compatible with our finding of a mean gaze following latency of 463 ms ($t_{follow}^{RJA}$, see Table 1 and Figure 2) when responding to a JA bid, Caruana et al. (2017, 2018) report median gaze following latencies of ~430 and ~ 465 ms, respectively, during a social situation (as compared to 300ms in response to non-social cues). Furthermore, a recent study of human-robot interaction (Willemse et al., 2018) demonstrated a similar latency (485 ms) for responding to robot gaze cues (after sustained experiences of JA), albeit a button press was used as a proxy for a gaze cued reaction in this study. Further, Pfeiffer et al. (2012) found that a latency of 400–800 ms for gaze following was perceived as being most “interactive.”

For non-responded JA bids, our object DT ($t_{back}^{IJA}=$ 1.590 ms) are shorter than those found by Pfeiffer-Leßmann et al. (2012) who reported empirical mean DTs of 1,900 ms. However, in Pfeiffer-Leßmann et al. (2012) the agent never responded to the JA bids but instead humans were instructed to look at the object until they felt the agent should have responded. Since we measured this parameter in non- responsive trials during an ongoing interaction of otherwise often successful JA bids, our results much more likely reflect the natural behavior during continuous interaction. Accordingly, DT of 1,200 and 1.800 ms were most likely perceived as intentional by humans in the study by Pfeiffer-Leßmann.

Interestingly, after successfully initiating JA (i.e., after the agent's gaze was also fixating the object) the time for a saccade back to the face was considerably lower in our study ($t_{object}^{RJA}=$ 888 ms) than in other studies (e.g., Bayliss et al., 2013; Willemse et al., 2018). Both studies used fairly restricted experimental tasks, whereas our paradigm created a continuous interactive experience. Bayliss et al. used an implicit gaze leading task with merely implied interaction, however, latencies were shorter for those conditions when a face followed the gaze of the human (as compared to incongruent gaze). Similarly, Willemse et al. (2018) found that saccade latency considerably decreased with the number of JA instances experienced with a robotic agent (1,500 vs. 1,100 ms). Accordingly, we also observed much longer latencies (in comparison to the JA condition) for back-to-face saccades if the agent did not respond with JA ($t_{back}^{IJA}>t_{object}^{IJA}$). Taken together, these findings suggest that social referencing (i.e., refocusing attention on a social partner) (Feinman et al., 1992; Bayliss et al., 2013; Willemse et al., 2018) is particularly enhanced during instances of continuous JA (i.e., shorter latencies for saccades that return to the face of the virtual character), lending credence to the immersiveness of the JA experience as evoked in our implementation. A further interesting aspect is the considerable amount of fixations on the agent's face we observed during the state. Even in the absence of any attempt to interact and despite explicit instruction to focus on objects (see also Bayliss et al., 2013), the presence of an agent with direct gaze still captures much of the human's attention (see e.g., Senju and Hasegawa, 2005).

To conclude, our results on temporal parameters of human gaze behavior are well in accordance with previous studies. Furthermore, our approach of an algorithmic implementation within a theoretical model of social gaze allows for substantial extensions of previous findings: Using the toolbox, agents and their parameters can be defined and varied according to the needs of specific interactions settings and experimental contexts, allowing for systematic and fine-grained exploration of social gaze behavior in a virtual environment. For example, we recently applied this toolbox to study the inference of communicative intent from gaze cues. To this end, we systematically manipulated the interactive states of an agent to determine differential gaze patterns of participants and their impact on the perception of deliberate communicative intent (Jording et al., 2019). Our virtual agent tool also has a high potential to be used for more complex scenarios beyond the “Social Gaze Space”. A range of complex social-emotional states could be conceived by recombining the building blocks of social gaze behavior, adding expressions of and sensitivity to facial emotions, and defining respective virtual agent behavior.

### 4.2. Applications

#### 4.2.1. Naturalistic Human-Agent Interaction

Advances in the technical development and computational power sparked the emergence of social, algorithmically controlled agents for face-to-face interactions. Such agents are increasingly used in diverse contexts, for example as assistants for “customer relations” (e.g., Kopp et al., 2005; Heaven, 2018), in interactive teaching contexts (e.g., Lee et al., 2015; Mabanza, 2016), and basic scientific research (e.g., von der Pütten et al., 2010; Courgeon et al., 2014; Grynszpan et al., 2017; Jording et al., 2019); for a general review on social robots see Mavridis (2015) and for more examples see Hoekstra et al. (2007), Pfeiffer et al. (2011), Gratch et al. (2013), Courgeon and Clavel (2013), and Pelachaud (2015). Compared to these approaches, our framework is focused on the conceptual framework of Social Gaze States (Jording et al., 2018) which provides a situation-specific taxonomy and exhaustive description of a specific behavior of interest. Moreover, it allows for the empirical determination of temporal parameters in a simple and highly controlled environment and provides the ability to add further adjustments to the agent's behavior. While this approach is tailored to a specific concept of gaze-based interaction, our results may still enrich these other approaches for human-agent interaction. The design of artificial agents requires an understanding of the underlying cognitive architectures incorporating natural JA behavior for action coordination (Deák et al., 2001). This includes the production of “natural” behavior which can be perceived as intentional by the human, but at the same time also a real-time prediction of the human's intentions based on his behavior. Both inference and display of intentional JA behavior can only be achieved with sufficient knowledge about the pattern and temporal “fine-tuning” of human reference behavior. The incorporation of such knowledge may greatly increase acceptance of artificial agents. For example, Huang and Thomaz (2011) found that robots which exhibit joint attention behavior during interactive tasks were consistently judged as performing better and their behavior was perceived as more natural.

In this respect, the present framework could be a valuable tool to define respective JA situations, their affordances, and determine the respective probabilistic and temporal parameters during real-life human-agent interaction. This is an essential step for the construction of realistic artificial agents for any kind of system for human interaction (e.g., Pfeiffer-Leßmann and Wachsmuth, 2009; Yu et al., 2010, 2012; Grynszpan et al., 2012; Stephenson et al., 2018; Willemse et al., 2018).

#### 4.2.2. Psychiatric Conditions

Increasingly, many psychiatric conditions have been conceived as disorders of social cognition (e.g., Crespi and Badcock, 2008; Vogeley and Newen, 2009; Moutoussis et al., 2014). Accordingly, several studies report deviations in gaze behavior during face-to-face situations, for example in social anxiety (Weeks et al., 2019), depression (Grossheinrich et al., 2018), schizophrenia (Caruana et al., 2019), and most prominently in autism spectrum conditions (ASC) (Frazier et al., 2017). In ASC, impairments in establishing JA are one of the earliest signs of a deficit in social communication (Mundy, 2003; Mundy and Newell, 2007) and subtle alterations can still be detected in adolescence (Oberwelland et al., 2017). Standardized behavioral assessment of JA, however, is available only for young children. Although preliminary studies in children and adolescents with ASC that use an unrestricted real-life setting do reveal differences in temporal patterns of gaze following, more fine-grained behavioral investigations including eye-tracking are urgently needed. More controlled settings which provide sufficient immersiveness, as implemented, would be ideal for further investigations. The present framework offers full control over the parameters governing gaze behavior of the agent and allows for in-depth assessment of temporal gaze patterns of the human to differentiate between specific states of attention and dynamic markers of on-going communication.

A further interesting application would be the implementation of agents displaying gaze patterns that resemble the behavior of persons under different psychiatric conditions (e.g., ASC). This could be used to investigate communicative behavior in a dyad with typical humans or in dyads of individuals with specific psychiatric conditions (Roth et al., 2020). Furthermore, prototypical gaze parameters for psychiatric conditions could be used as diagnostic markers and to define training targets for interventional studies.

#### 4.2.3. Limitations

Due to the usage of prerendered 2D images for the agent, only predefined facial expressions and gaze directions can be displayed. To reduce complexity we also decided to focus on pure eye-gaze shifts without head-rotation, thus other potentially important aspects such as head rotation velocity, eye-head shift ration could not be investigated. Therefore, our visual presentation may offer less immersiveness than such approaches using real-time 3D rendering (Linowes, 2015). On the other hand, our approach offers through the usage of PsychoPy a highly controlled and stimulus sparse experimental environment with highly reliable stimulus presentation times. Furthermore, it should be possible to migrate the present Python code module for agent behavior into other experimental frameworks using different approaches for visual presentation. The current version of the toolbox is tailored to a specific concept of joint attention (*Social Gaze States*). Although our implementation is flexible and can accommodate other scenarios, the implementation of further scenarios will require exact definitions of respective behavioral constraints and algorithmic implementations. Similarly, although we provide the technical implementation of eye blinks and responsiveness to facial expressions of emotion, future studies and extensions of the theoretical concept are needed to investigate the coordination of such behaviors and joint attention. Furthermore, we make the assumption that the dynamics of our specified macro states are generic, i.e., comparable across participants. This might be the case for the specified macro states here, but may not hold for more complex scenarios. Research into variability and individual differences will be necessary in these cases.

## 5. Conclusion

The present work aims at encouraging in-depth exploration of patterns in human social gaze behavior and their dynamics: We present a virtual agent that embodies the *Social Gaze States* (Jording et al., 2018) and demonstrate how such an implementation can be used to iteratively determine the temporal and probabilistic dynamics of human gaze behavior during gaze-based interaction. The modular and extendable structure of the framework offers a flexible approach to create highly controlled experimental virtual environments for social gaze research with potential applications in the investigation of psychiatric conditions and naturalistic human-agent interaction. It demonstrates flexibility for implementing behavioral states of virtual agents in instances of joint attention for the creation of individual paradigms along with technical reliability and may spark further research and insights into the dynamics of social gaze interaction.

## Data Availability Statement

The data will be made available upon request, the code for creating experiments is available at https://github.com/arnohakk/TriPy.

## Ethics Statement

The studies involving human participants were reviewed and approved by Ethics Committees of University Hospital Cologne and University Hospital RWTH Aachen. The participants provided their written informed consent to participate in this study.

## Author Contributions

AH and BG designed and implemented agent behavior and data recording. MJ collected the data. AH and MJ implemented data analysis. MS-R supervised software development and data analysis. AH drafted the manuscript. BG, MJ, KV, and MS-R revised it critically. All authors contributed to the article and approved the submitted version.

## Conflict of Interest

The authors declare that the research was conducted in the absence of any commercial or financial relationships that could be construed as a potential conflict of interest.

## Publisher's Note

All claims expressed in this article are solely those of the authors and do not necessarily represent those of their affiliated organizations, or those of the publisher, the editors and the reviewers. Any product that may be evaluated in this article, or claim that may be made by its manufacturer, is not guaranteed or endorsed by the publisher.
