# Supplementary material for: Temporal Behavioral Parameters of On-Going Gaze Encounters in a Virtual Environment
Source: Front Psychol. 2021 Aug 6;12:673982. doi: 10.3389/fpsyg.2021.673982 (PMC8377250; doi:10.3389/fpsyg.2021.673982)
Supplement: Supplementary file 6 [file Data_Sheet_1.pdf]

Supplementary Material for Temporal Behavioral Parameters of on-going Gaze Encounters in a Virtual Environment

1 SUPPLEMENTARY METHODS

1.1 Formal definition of agent behavior

Each macro state M is a set of n_M micro states:

$$M = \{x_1^M, \ldots, x_{n_M}^M\} \tag{S1}$$

Each micro state is defined by a set of characteristics describing its appearance to the participant, its duration, and transition probabilities to other micro states within the macro state:

$$x = \{a_x, \boldsymbol{\tau}_x^p, \boldsymbol{p}_x^p, \boldsymbol{\tau}_x^i, \boldsymbol{p}_x^i\} \tag{S2}$$

1. a_x: nonverbal appearance of the agent conveying social information to the participant (e.g. gaze direction).
2. $\boldsymbol{\tau}_x^p$: a set of random distribution q_x^p and corresponding parameters $\Theta_x^p = \{\mu_x^p, \sigma_x^p, \ldots\}$ to draw a micro state duration $d_x^p = f(\tau_x^p) = q_x^p(\Theta_x^p)$. [1] The index p denotes that these parameters are for passive, i.e. non-interactive, micro state transitions.
3. $\boldsymbol{p}_x^p = (p^p(x_1|x), p^p(x_2|x), \ldots, p^p(x_n|x))$ with $\sum_i p^p(x_i|x) = 1$ and $p(x_i|x_i) = 0$: a vector containing probabilities for transitioning passively from micro sate x to another micro state x_k with $k \in (1 \ldots n)$ after state duration d_x^p.[2]
4. $\boldsymbol{\tau}_x^{s^i}$: Same as $\boldsymbol{\tau}_x^p$, but for determining micro state duration $d_x^{s^i}$ after registration predefined socio-emotional signal s^i of the participant (i.e. fixations on AOIs) to which micro state x is sensitive.
5. $\boldsymbol{p}_x^{s^i}$: Same as $\boldsymbol{p}_x^p$, but again for an socio-emotional signal s^i as in 4.

According to this definition, there are two different possibilities for micro state transition: Either (1) interactively in response to a behavioral event s_i emitted by the participant after a specified post-event time $d_x^{s_i} = q_x^{s_i}(\Theta_x^i)$ (top arrows in Fig. S1) or (2) passively after a micro state duration $d_x^p = q_x^p(\Theta_x^p)$ (bottom arrows in Fig. S1). In this latter case, agent behavior resembles a simple Markov chain as the next micro state x_{t+1} only depends on the current state x_t via transition probabilities $\boldsymbol{p}_{x_t}^p$.

To the participant only the obvious presentation of non-verbal appearance a_x and the actual micro state duration d_x, but not the dependencies and underlying distributions are directly observable.

Please note, that the aim of this study is to estimate temporal and probabilistic parameters from empirical data described as part of the method section 2.2.3 of the main text: Each $\boldsymbol{\tau}$ is estimated from a timing parameter as depicted in Fig. 2.

[1] An example would be being a Gaussian distribution and Θ being variance σ and mean μ.

[2] $p(\cdot|\cdot)$ reads as a conditional probability: $p(x_j|x_k)$ is the probability of transitioning to micro state x_j given that the agent is in state x_k.

1.2 Supplementary Hard- and Software Information

1.2.1 Software

The visual representation of the agent's behavior is generated by switching between pre-rendered images displaying changes in gaze direction and/or facial expression. They were created with DAZ Studio 4.9 (DAZ Productions, Inc., USA), a software package which is freely available and creates detailed and highly configurable facial expressions with sufficient objective realism while providing an easy to use interface. The virtual character representing the agent to the participant was selected from an online survey of multiple possible virtual characters meeting the criteria of an average rating for dominance and trustworthiness ((Oosterhof and Todorov, 2008) (rated 5.2 ± 2.2 and 5.3 ± 1.6, respectively on a a nine-point likert scale ($0 =$ characteristic not shown, $9 =$ very strongly shown) in a sample of twenty-seven participants. Within the present framework, virtual characters can be easily changed to meet the requirements for experimental manipulation.

1.2.2 Hardware

This framework was implemented using a Tobii TX300 eye tracker (Tobii Technology, Sweden), which allows for head movement within a tracking box of 30 * 37 * 17 cm^3 at a sampling rate of $300\,\mathrm{Hz}$. This relatively unrestrained setting (i.e. no need for a chin rest) supports the impression of a natural interaction and increases ecological validity especially for social encounters. Other eye tracking systems could be added with minimal/reasonable effort if they are supported by PyGaze.

A standard "off-the-shelf" webcam with reasonably good image quality can be used for synchronized video recording of the participant via μ Cap (Doyle and Schindler, 2015).

The framework was developed and tested on contemporary mid range performance PC hardware (see Tab. S5 for a detailed lists of systems). Compatibility to Linux was only briefly tested on a slow machine (Lenovo ThinkPad x230) and no software issues occurred.

For offline analysis and more accurate stimulus presentation timings on the computer screen, support for Cedrus StimTracker (Cedrus Corporation, USA) connected to the TX300 is implemented as an optional feature.

1.2.3 Technical Reliability

Since our toolbox adds a rather big overhead to PyGaze, accurate monitoring and minimization of any possible latencies in the availability of eye tracking data was of utmost importance throughout the development. This is particularly important for gaze-contingent paradigms because inaccuracies are a source of noise in experimental data and might disrupt the smooth flow of the experiment and the experience of natural interaction. To ensure the accuracy of measurements, the timestamp of the last available gaze data package (from the eye tracker, as provided by the Tobii SDK) was compared to the CPU time at the moment of its acquisition. Due to the format of gaze data from Tobii eye trackers (time variable with an arbitrary starting point), it is not possible to give a reliable estimate of this lag during run time, only a jitter in acquisition. The present framework introduces uncertainties at least two magnitudes smaller than the effects of interest in social gaze research (1 to 100's ms) (Fig. S6).

1.2.4 Availability and Documentation

The code is available via https://github.com/arnohakk/TriPy and comes with a example paradigm and annotated code to give an interested user a first impression and the possibility to easily adapt and extend

the code. Included features experimental features and behavioral parameters can be adapted via simple configuration files. For more details, see the README file under above link.

1.3 Supplementary Experimental Methods

1.3.1 Participants

37 adult volunteers (20 identifying themselves as female, 16 as male, 1 as queer; mean age 28.2 $\pm$ 9.2) with no self-reported record of neurological or psychiatric conditions took part in the experiment. The study was approved by the Research Ethics Committees of University Hospital Cologne and University Hospital RWTH Aachen. All volunteers were recruited via mailing lists and postings on the campus of the University of Cologne and gave written informed consent prior to participation. All data was acquired in Cologne.

1.3.2 Data (pre)processing and exclusion

Data analysis was performed using R v3.4.4 (R Core Team, 2018) on Ubuntu 18.04.

1.4 Supplementary Data Analysis Methods

1.4.1 Fixation/saccade classification

For offline gaze event detection (fixations and saccades) the raw eye tracking data was smoothed in a sliding window procedure to reduce the impact of noise before classifying fixations and saccades based on an algorithm proposed by (Engbert et al., 2002) as implemented in the analysis pipeline for a freely-moving head.

This choice was made to keep on- and offline results as similar as possible: An adapted version of this algorithm for real time saccade detection is implemented in PyGaze, which should be favored according to the author (Dalmaijer et al., 2014) over the one for fixation detection for gaze-contingency. Unfortunately, the PyGaze saccade detection algorithm is not robust against head movements and the present framework relies on its fixation detection routines which appeared to be robust.

1.4.2 AOI definitions

Fixations were assigned to user-defined AOIs (Fig. S5). Generally, arbitrary AOI shapes are possible as they are defined via geometric shapes of the (Bivand et al., 2013) package.

1.4.3 Heat maps

For each induced gaze state, we constructed logarithmized heat maps for illustration by drawing a circle around each mean fixation position (x, y) with a radius of $max(SD(fix_x), SD(fix_y))$. This circle was convoluted with a quadratic density distribution around the center integrating to a value proportional to the duration of the fixation. The resulting map was logarithmized due to high fixation density in the eye region and overlayed on a screen shot of the paradigm.

REFERENCES

Bivand, R. S., Pebesma, E., and Gomez-Rubio, V. (2013). *Applied spatial data analysis with R, Second edition* (Springer, NY)

Dalmaijer, E. S., Mathôt, S., and Van der Stigchel, S. (2014). Pygaze: An open-source, cross-platform toolbox for minimal-effort programming of eyetracking experiments. *Behavior Research Methods* 46, 913–921. doi:10.3758/s13428-013-0422-2

Doyle, L. and Schindler, D. (2015). *muCap: Connecting FaceReader^{TM} to z-Tree*. Discussion Papers in Economics 24809, University of Munich, Department of Economics

Engbert, R., Longtin, A., and Kliegl, R. (2002). A dynamical model of saccade generation in reading based on spatially distributed lexical processing. *Vision Research* 42, 621 – 636. doi:https://doi.org/10.1016/S0042-6989(01)00301-7

Hessels, R. S. (2017). Toward early markers for autism spectrum disorder using eye tracking. *Journal of Cognitive Education and Psychology* 16, 310–312

Oosterhof, N. N. and Todorov, A. (2008). The functional basis of face evaluation. *Proceedings of the National Academy of Sciences* 105, 11087–11092. doi:10.1073/pnas.0805664105

R Core Team (2018). *R: A Language and Environment for Statistical Computing*. R Foundation for Statistical Computing, Vienna, Austria

2 SUPPLEMENTARY FIGURES

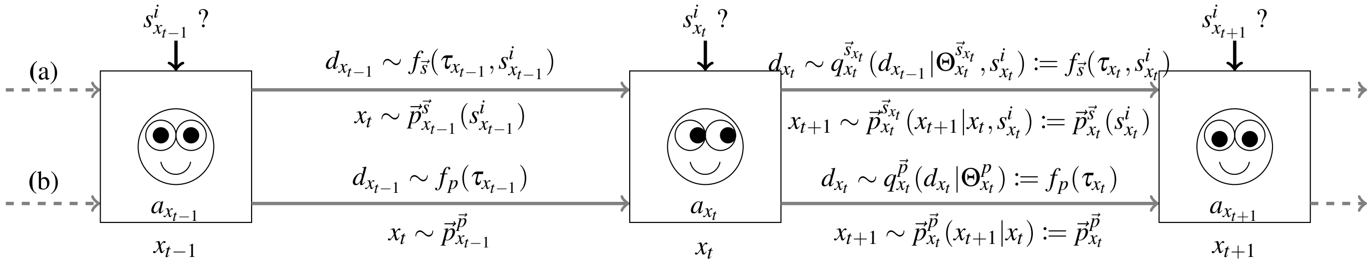

Figure S1. Snapshot from algorithmically generated micro state chain of an agent changing its gaze direction. Micro state transition can occur either **(a)** interactively in response to a participant's socio-emotional signal $s^i_x \in \boldsymbol{S}_x$ to which micro state x is sensitive (e.g. fixation on AOI, target facial expression classification) or **(b)** non-interactively via a Markovian process. $d_x \sim q^\alpha_x(d_x|\Theta_x(, S^i_x))$ denotes that micro state duration d_x is sampled from a state-defining random distribution q_x with a corresponding set of parameters Θ_x, for interactive ($\alpha = \boldsymbol{s}$) and passive state transitions ($\alpha = \boldsymbol{p}$) respectively. The short notation $q^\alpha_x(d_x|\Theta_x(, s^i_x)) := f_\alpha(\tau_x(, s^i_x))$ denotes that the duration d_x is a function of the set $\tau_x = \{q_x, \Theta_x\}$ of the distribution q_x and corresponding parameters $\Theta_x = \{\mu_x, \sigma_x, \dots\}$, and in the interactive case, also of the detected socio-emotional signal s^i_x.

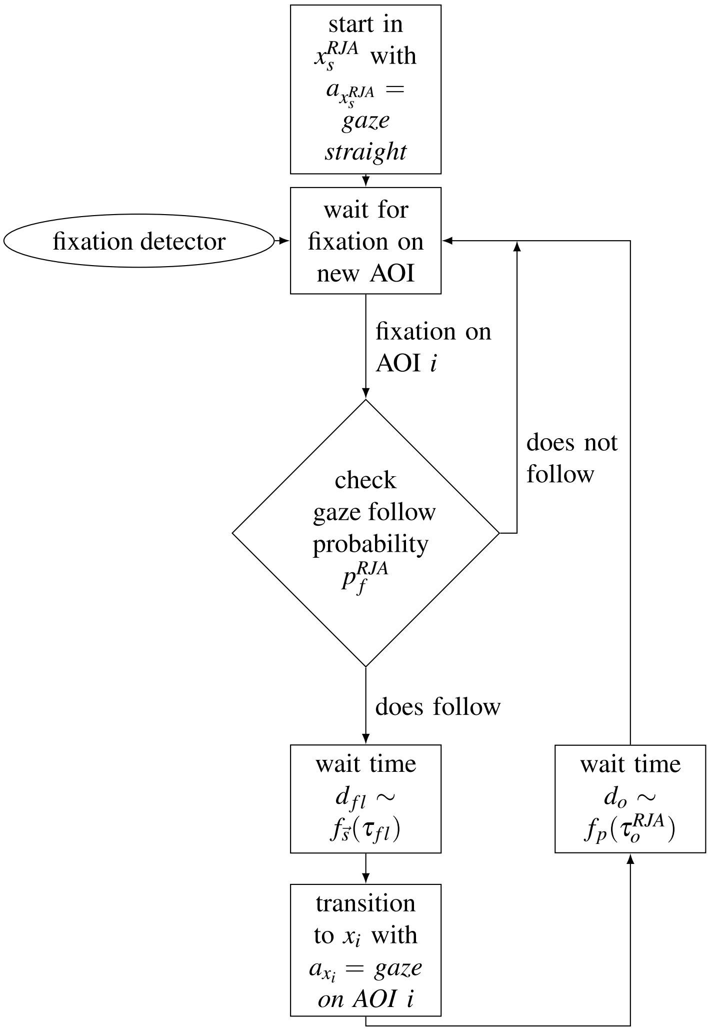

Figure S2. State diagram: RJA Agent. The agent starts gazing straight at the participant waiting for a fixation on one of the predefined AOIs. With follow probability p_f, it changes to micro state x_i with a_x =gaze on AOI i. After a time $d_o \sim f_p(\tau_o^{RJA})$, the agent waits again for a fixation on a new AOI, ready to follow another JA bid by the participant. Note, that the agent was also blinking with the eyes during the interaction with the human.

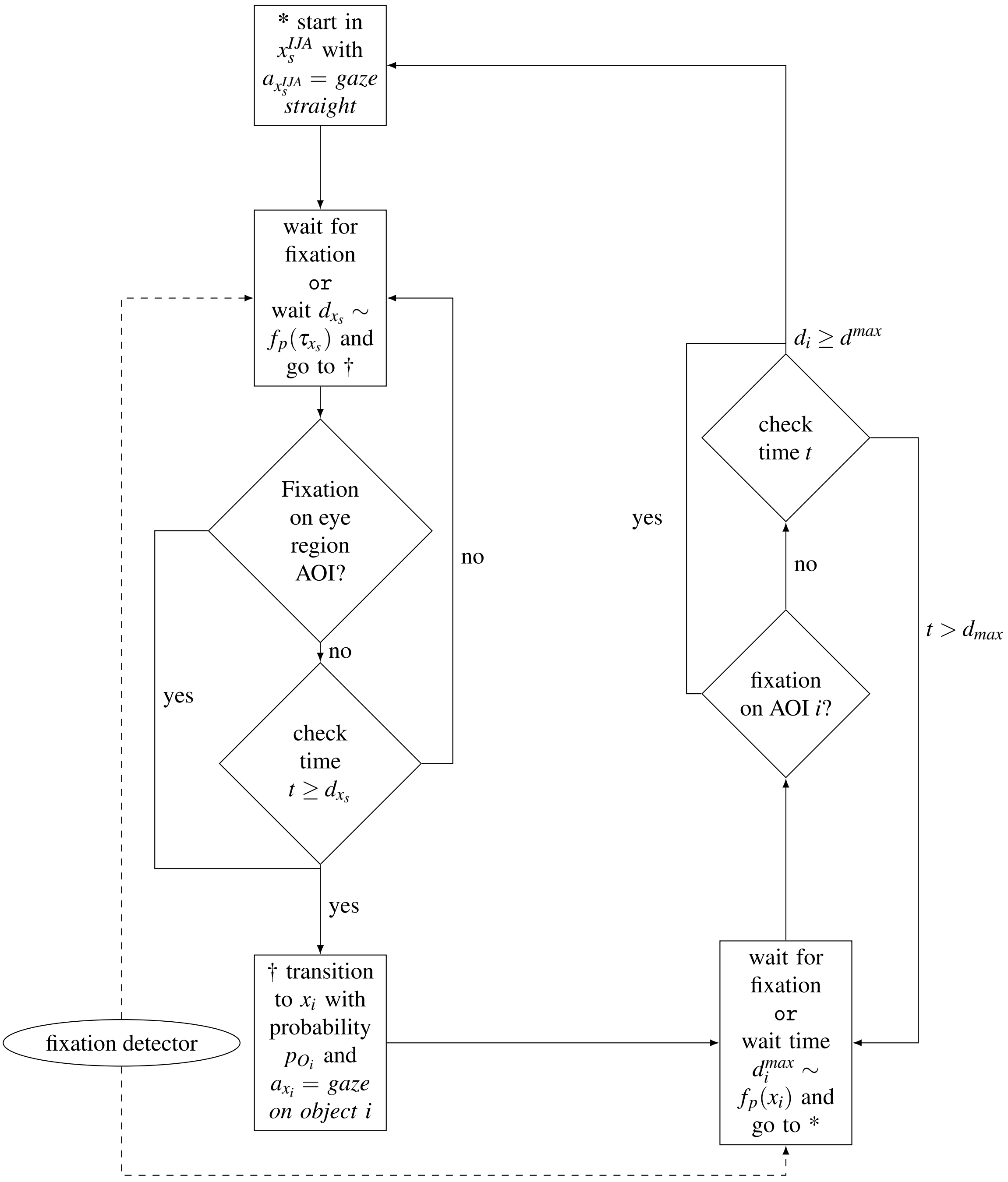

Figure S3. State diagram: IJA macro state. The agent starts in micro state x_s^{IJA} gazing straight at the participant trying to establish eye contact. After eye contact is established, or after a waiting time t, the agent tries to initiate JA on object i, i.e. it transitions to micro state x_i, with probability p_{O_i} and gaze on object i. It then waits for a time t for a fixation by the participant on the same AOI i, before switching back to micro state x_s and starts a new JA bid. Note that in this case, micro state transitions can either be passive or interactive

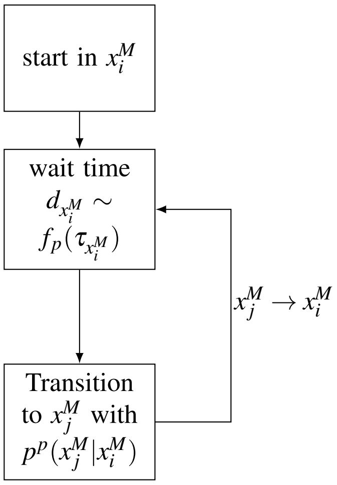

Figure S4. State diagram: *Non-interactive Agents* ($M \in \{PO, OO, INT\}$). The agent starts in a micro state x_i^M and waits for a state duration $d_{x_i^M} \sim f_p(\tau_{x_i^M})$ before transitioning to a new micro state x_j^M with probability $p^p(x_j^M|x_i^M) \in \boldsymbol{p}_{x_i}$, and samples again new micro state duration. This resembles the type of state transitions depicted by the bottom arrows in Fig. S1.

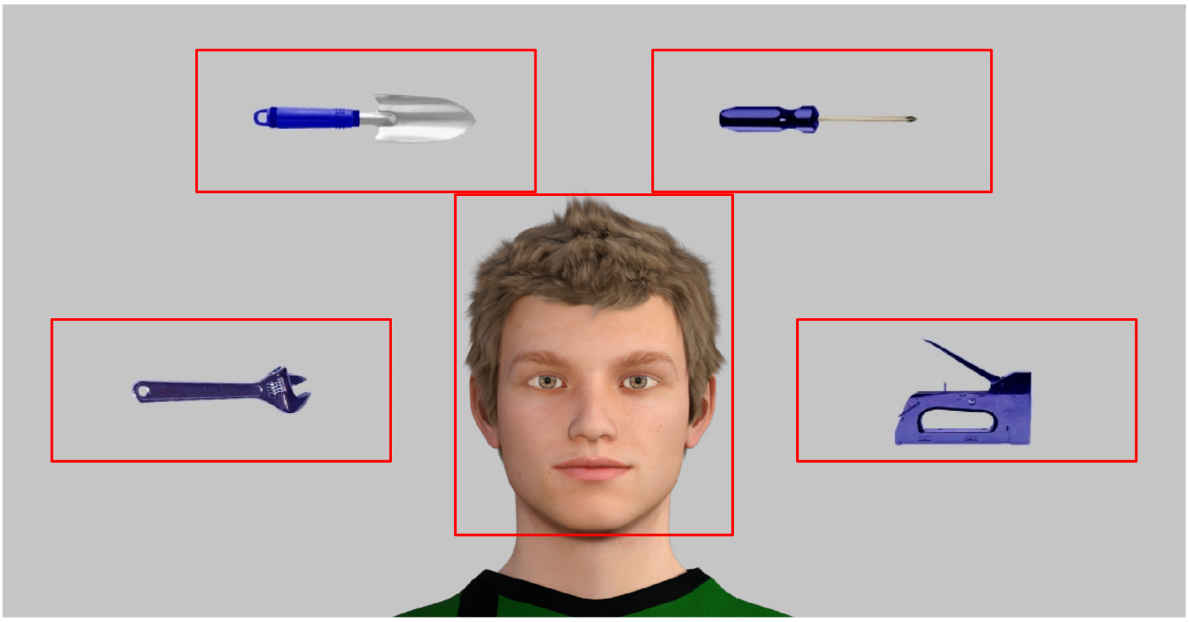

Figure S5. AOIs (red rectangles) for offline data analysis to estimate behavioral parameters. Slightly larger-than-object-size AOIs were used for noise robustness (Hessels, 2017)

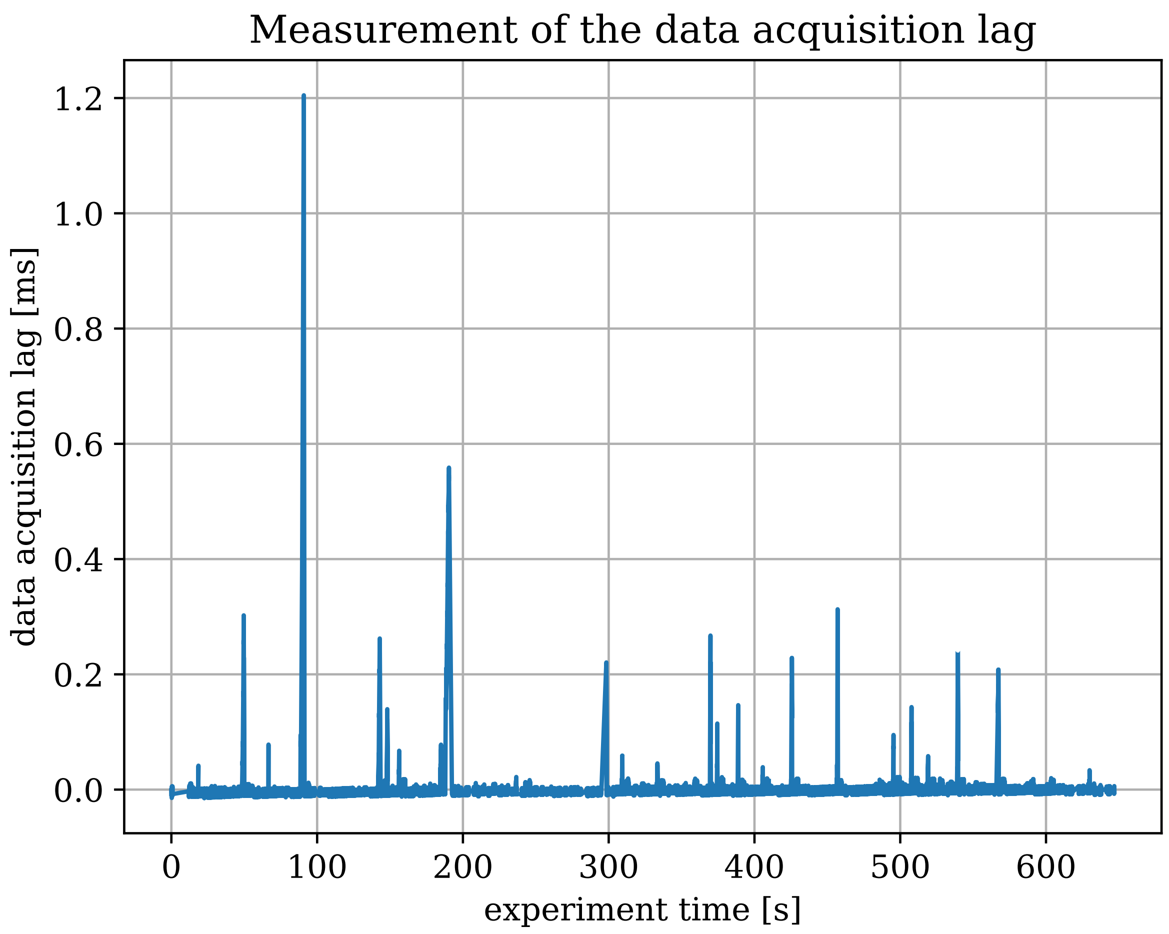

Figure S6. Difference between the system clocks of the eye tracker (as provided by the Tobii SDK) and stimulus PC over the course of one experimental block. Spikes indicate a neglectable lag ($\sim 1\,\mathrm{ms}$) in the acquisition of data from the eye tracker by the PC clock.

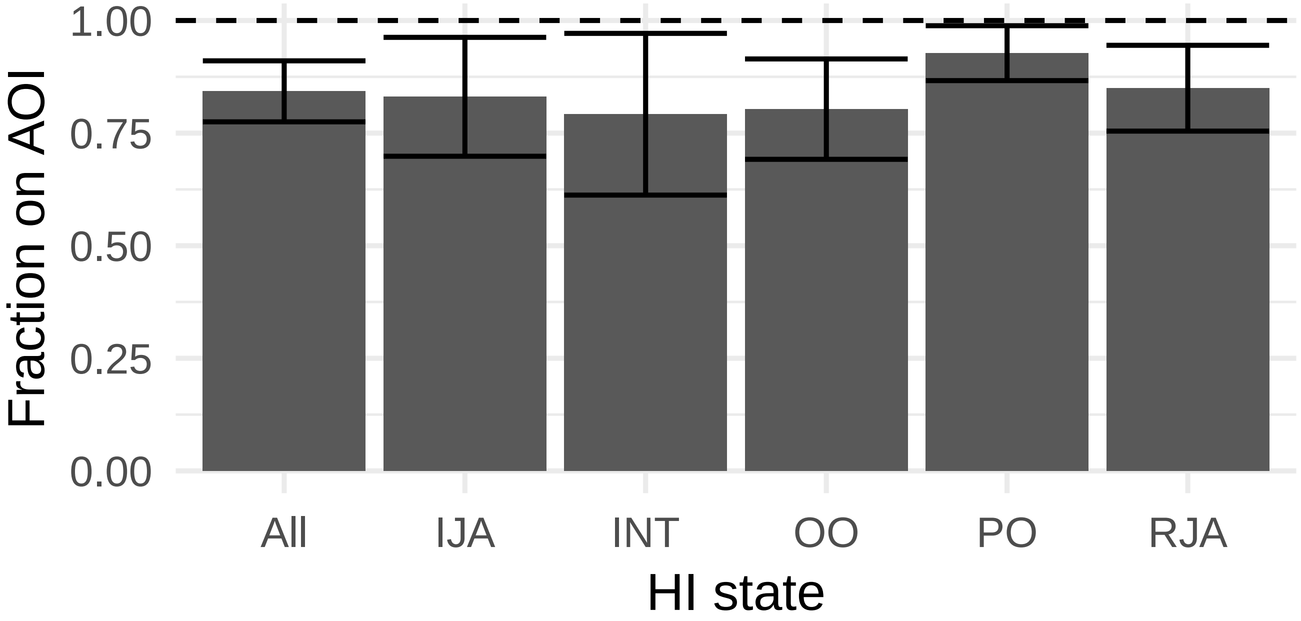

Figure S7. On-Aoi ratio r_{aoi}^{M} across participants.

3 SUPPLEMENTARY TABLES

Table S1. Interactive macro state: RJA. Each micro state $x_k^{RJA} \in RJA$ is defined via its appearance a_{x_k}, a follow probability $p_{f_{x_k}}$, and temporal parameter sets $\boldsymbol{\tau}^{\alpha}_{x_k^{RJA}}$ of a probabilistic distribution $q^{\alpha}_{x_k^{RJA}}$ and corresponding parameters $\Theta^{\alpha}_{x_k^{RJA}} = \{\mu^{\alpha}_{x_k^{RJA}}, \sigma^{\alpha}_{x_k^{RJA}}, \dots\}$ for drawing state durations $d_{x_k^{RJA}} \sim q^{p}_{x_k^{RJA}}(d_{x_k^{RJA}} | \Theta_{x_k^{RJA}})$ as depicted in Fig. S1.

micro state	a_{x_k}	set	description
$\{x_k^{RJA}\}$	gaze on AOI k (either on an object or straight at the participant)	$p^{RJA}_{f_{x_k}}$	Probability of agent following on same AOI for successful JA
		$\boldsymbol{\tau}^{follow}_{x_k^{RJA}}$	Time after which agent will follow with gaze on AOI for successful JA
		$\boldsymbol{\tau}^{object}_{x_k^{RJA}}$	Time agent keeps fixating object after successful JA and participant not fixating AOI anymore

Table S2. Interactive macro state: IJA. Notation analog to Tab. S1. Note that all states have both passive and interactive possibilities for state transitions.

state	a_x	set	S	description
$x^{IJA}_{straight}$	gaze straight (on participant)	$\boldsymbol{\tau}^{i}_{x^{IJA}_{straight}}$	*gaze on AOI* i	Duration of the agent looking straight after detected fixation on agent's eyes AOI before fixating an object to try to initiate JA.
		$\boldsymbol{\tau}^{p}_{x^{IJA}_{straight}}$	-	Duration of the agent looking straight at the participant if no fixation on the eyes AOI was detected before choosing an AOI to gaze at to try to initiate JA.
		$\boldsymbol{p}^{p,i}_{x^{IJA}_{straight}}$	-	Transition probabilities to micro states $\{x^{IJA}\}$.
$x^{IJA}_{object_i}$	gaze at AOI i (object i)	$\boldsymbol{\tau}^{i,IJA}_{x_{object_i}}$	*fixation loss*	Duration of the continuation of the agent looking at the AOI on which in tried to initiate JA and after a fixation of the participant has been detected on that AOI.
		$\boldsymbol{\tau}^{p,IJA}_{x_{object_i}}$	-	Duration of the agent looking at the AOI on which it tried to initiate JA and if no fixation of the participant was detected on that AOI.
		$\boldsymbol{p}^{p,i}_{x^{IJA}_{o_{object_i}}}$	-	Transition probabilities to micro states $\{x^{IJA}\}$. With $p(x_s\|x_{object_i}) = 1$ it is ensured that the agent will always look straight after a round of (tried) IJA.

Table S3. Non-interactive macro states $M \in \{PO, OO, INT\}$. Notation analog to Tab. S1. Note that all states only have passive state transitions. The different appearance to the participant is created by using different numerical values for each state.

state	$a_{x^M_k}$	set	S	description
$\{x^M_k\}$	gaze at AOI k	$\boldsymbol{\tau}^{p}_{x^M_k}$	-	duration of micro state i
		$\boldsymbol{p}^{p}_{x^M_k}$	-	probabilities of transitioning from micro state x^M_k to any other micro state from $\{x^M_k\}$

Table S4. Instructions presented to participant to interact with the agent. For the sake of enhanced ecological validity the agent was named “Paul” in the instructions.

macro state	instruction (translated from German)
INT *introspective*	Please keep your eyes open and focus on your breath.
OO *object-oriented*	Please focus on the objects.
PO *partner-oriented*	Please focus on Paul.
RJA *responding JA*	Please interact with Paul and let him guide you.
IJA *initiating JA*	Please interact with Paul and try to guide him.

Table S5. PC hardware.

component	System 1	System 2
CPU	Intel Xenon E5-1620v2	Intel Xenon E5-1603v3
RAM	8 GB DDR3 1600 MHz	16 GB DDR4 3000 MHz
GPU	Nvidia Quadro K2000	Nvidia Quadro K420
OS	Windows 7	Windows 7

Table S6. Macro state combinations measured in 1st paradigm: The numbers show how often a given macro state combination was used in the paradigm. The $6 + 6$ for the RJA-IJA combination denotes that the RJA agent was used with two different follow probabilities. ($p_f^1 = 100\%$ and $p_f^2 = 33\%$). To keep the experiment as short as possible, we only used macro state combinations of interactive states that can lead to successful JA.

participant \ A	INT	OO	PO	RJA	IJA
INT	6	-	6	-	-
OO	6	-	6	-	-
PO	6	-	6	-	-
RJA	-	-	-	-	12
IJA	-	-	-	$6 + 6$	-

Table S7. Transition probabilities in INT state used paradigm.

aoi	down	down left	down right	straight
down	.3	.3	.3	.1
down left	.3	.3	.3	.1
down right	.3	.3	.3	.1
straight	.3	.3	.3	.1

Table S8. Transition probabilities in PO state used in paradigm.

aoi	down	down left	down right	straight
down	.033	.033	.033	.9
down left	.033	.033	.033	.9
down right	.033	.033	.033	.9
straight	.033	.033	.033	.9

Table S9. Temporal parameter sets used in paradigm. All distributions $\{q\}$ were set as Gaussians.

State	Parameter name	mean in ms	SD in ms
all	$\boldsymbol{\tau}_{time\ between\ blinks}$	3500	500
	$\boldsymbol{\tau}_{blink\ duration}$	100	0
RJA	$\boldsymbol{\tau}^{RJA}_{follow}$	500	100
	$\boldsymbol{\tau}^{RJA}_{object}$	500	150
IJA	$\boldsymbol{\tau}^{IJA}_{straight}$	1500	300
	$\boldsymbol{\tau}^{IJA}_{objects}$	500	100
	$\boldsymbol{\tau}^{IJA}_{back}$	6000	300
INT	$\boldsymbol{\tau}^{INT}_{k}$	2800	700
PO	$\boldsymbol{\tau}^{PO}_{k}$	2800	700

Table S10. Interactive probabilistic parameters used in paradigm.

State	set	value
IJA	$\{p^{p,i}_{x^{IJA}_{o_{object_i}}}\}$	.25
$RJA1$	$p^{RJA1}_{f_{x_k}}$	1.0
$RJA2$	$p^{RJA2}_{f_{x_k}}$	.33
